# Genetic Variation and Potential for Resistance Development to the tTA Overexpression Lethal System in Insects

**DOI:** 10.1534/g3.120.400990

**Published:** 2020-02-04

**Authors:** Katherine E. Knudsen, William R. Reid, Traci M. Barbour, Laci M. Bowes, Juliana Duncan, Elaina Philpott, Samantha Potter, Maxwell J. Scott

**Affiliations:** * Department of Biological Sciences, Program in Genetics,; † Molecular Biotechnology Training Program,; ‡ Department of Entomology and Plant Pathology, North Carolina State University, Raleigh, NC 27695

**Keywords:** DGRP, fs-RIDL, sterile insect technique, tetracycline transactivator, male-only strain

## Abstract

Release of insect pests carrying the dominant lethal tetracycline transactivator (tTA) overexpression system has been proposed as a means for population suppression. High levels of the tTA transcription factor are thought to be toxic due to either transcriptional squelching or interference with protein ubiquitination. Here we utilized the *Drosophila melanogaster* Genetic Reference Panel (DGRP) to examine the influence of genetic variation on the efficacy of a female-specific tTA overexpression system. The level of female lethality between DGRP lines varied from 11 to 97% with a broad sense heritability of 0.89. A genome-wide association analysis identified 192 allelic variants associated with high or low lethality (*P* < 10^−5^), although none were significant when corrected for multiple testing. 151 of the variants fell within 108 genes that were associated with several biological processes including transcription and protein ubiquitination. In four lines with high female lethality, tTA RNA levels were similar or higher than in the parental tTA overexpression strain. In two lines with low lethality, tTA levels were about two fold lower than in the parental strain. However, in two other lines with low lethality, tTA levels were similar or approximately 30% lower. RNAseq analysis identified genes that were up or downregulated in the four low female lethal lines compared to the four high lethal lines. For example, genes associated with RNA processing and rRNA maturation were significantly upregulated in low lethal lines. Our data suggest that standing genetic variation in an insect population could provide multiple mechanisms for resistance to the tTA overexpression system.

Each year, around 3 million tons of pesticides are used throughout the world (Pimentel 2009b). While this method may be effective, it is not without controversy, as concerns arise surrounding the risks associated with long-term pesticide use and its effects on both the environment and human health (Pimentel 2009a). Certain insecticides, which were once thought to be safe, cause unexpected environmental consequences, for example the weakening of eggshells of birds exposed to DDT (Pimentel 2009b, 2009a). Widespread use of synthetic insecticides appear to be one of the drivers responsible for the global decline of insects (Sánchez-Bayo and Wyckhuys 2019). Some chemical pesticides have been linked to human disorders such as Alzheimer’s Disease, Parkinson’s Disease, Attention Deficit Hyperactive Disorder and cancer (Baldi 2003; Betarbet *et al.* 2000; Kuehn 2010; Alavanja *et al.* 2004; Pimentel 2009a). Also of concern is the development of pest resistance to insecticides, causing farmers to use higher concentrations of chemicals or combinations of pesticides (Pimentel 2009a).

An environmentally safe approach to pest management is the Sterile Insect Technique (SIT), a successful method introduced by E.F. Knipling in the 1950s (Knipling 1955). SIT employs the release of insects sterilized by radiation into the area of the crops affected by the pest population. These sterile insects, released in high numbers, breed with the wild population but the sterile matings do not produce offspring. With repeated releases over time, the wild population dwindles, resulting in either eradication of the pest population or a drop to more manageable population numbers. This method has proven successful in the eradication of the New World screwworm, *Cochliomyia hominivorax*, from North America (Klassen and Curtis 2005; Scott *et al.* 2017), as well as with management of the Mediterranean fruit fly, *Ceratitis capitata* (Rendón *et al.* 2004), and the Mexican fruit fly, *Anastrepha ludens* (Schetelig *et al.* 2016).

Classic SIT utilizes high doses of ionizing radiation to sterilize insects for release, but this radiation may affect the fitness of treated insects (Klassen and Curtis 2005). SIT is more efficient if only males are released (Rendón *et al.* 2004). To this end genetic sexing strains (GSSs) were developed for the Mediterranean fruit fly that permit the mass release of sterile males (Rendón *et al.* 2004). However, development of GSSs took many years, as the strains carry recessive selectable mutations (white pupae, embryo temperature sensitive lethal) and stable Y-linked translocations with the wild type allele. Genetically engineered systems can involve transmission of dominant lethal genes into the pest population that eventually lead to suppression of the population or genes that transform or replace a vector population with insects that cannot transmit disease (Horn and Wimmer 2003; Alphey 2014). Each method requires the release of transgenic insects into the field to breed with the pest population, leading to suppression or replacement of the wild pest species. However, epistatic modifiers in the genetic background of the pest species may cause each developed transgenic strain to differ in fitness and efficiency of the control system. That is, there is a potential for development of resistance to lethal transgenes or genes that disrupt transmission of disease. Innovation in assessing and minimizing the potential risks of genetic pest management systems will ensure that any method employed in the field will use transgenic insects of the highest efficiency and fitness of the candidate transgenic strains, thereby ensuring that the system remains effective and environmentally friendly.

Oxitec’s Release of Insects carrying a Dominant Lethal (RIDL) system, a modified version of SIT, has been developed for genetic control of *Aedes aegypti* and other pests (Fu *et al.* 2010; Atkinson *et al.* 2007; Alphey and Andreasen 2002; Alphey *et al.* 2010; Black *et al.* 2011; Carvalho *et al.* 2015). *Ae aegypti* is the mosquito vector of dengue, yellow fever and Zika viruses (Carvalho and Moreira 2017). The *Ae. aegypti* strain carries a simple genetic system consisting of the tetracycline transactivator gene (tTA) with a core promoter and multiple binding sites for tTA (Phuc *et al.* 2007). In the absence of tetracycline, tTA is overexpressed leading to lethality at late larval/pupal stages. Death is thought to be due to “transcriptional squelching,” which is a general interference in gene expression (Gong *et al.* 2005). The *Ae. aegypti* strain developed had incomplete penetrance, with 3–4% of the offspring from crosses with the non-transgenic parental strain developing to adults on diet that lacked tetracycline (Phuc *et al.* 2007). The incomplete penetrance could explain the observed introgression of some of the transgenic strain genome into a targeted natural population in Brazil (Evans *et al.* 2019). A variation of this system, fsRIDL, was developed for *C. capitata* that can produce only males on diet without tetracycline as the *tTA* gene contains the sex-specific intron from the C. capitata transformer (*tra*) gene (Fu *et al.* 2007). Some of the lines showed high female survival (over 50%) with one copy of the transgene but were 100% female lethal when homozygous with two copies. This suggests that small changes in gene expression (*e.g.*, twofold) could significantly influence the level of female lethality.

We previously developed a sex-specific conditional lethal system in *Drosophila melanogaster* that is similar to the *C. capitata* system (Li *et al.* 2014). In our FL3 system, 21 copies of the tetO enhancer site are linked to the Drosophila *hsp70* core promoter and the tTA gene sequence is interrupted by the sex-specific intron from the *C. hominivorax tra* gene (Supplementary Figure 1). In *D. melanogaster*, the Sex lethal (SXL) protein regulates splicing of *tra*, but in *C. hominivorax*, tephritid fruit flies and other flies, *tra* alternative splicing is autoregulated (Verhulst *et al.* 2010). The sex-specific *tra* intron contains multiple predicted TRA/TRA2 binding sites (Li *et al.* 2013). In *C. hominivorax*, the shorter female transcript encodes functional TRA protein whereas the longer male transcripts include an additional exon with multiple in-frame translation stop codons (Li *et al.* 2013). The male transcript encodes a much shorter protein that is likely nonfunctional. In *D. melanogaster* carrying the FL3 transgene the tTA transcript is correctly sex-specifically spliced, suggesting that the *C. hominivorax tra* intron TRA/TRA2 sites are functional in Drosophila. Thus, when reared in the absence of tetracycline, homozygous females carrying a FL3 transgene died mostly at the pupal stage(Li *et al.* 2014). Female lethality was 100% for homozygotes and approximately 90% for heterozygotes. The FL3 transgenic lines were made in an isogenic Canton-S-B strain to minimize variation in the genetic background. The FL3 system was also functional in the Australian sheep blowfly *Lucilia cuprina* (Li *et al.* 2014). Similar to what was observed in *C. capitata* (Fu *et al.* 2007), females of one line showed high survival with one copy of the transgene but with two copies only males were produced on diet without tetracycline (Li *et al.* 2014).

*D. melanogaster* is not an economic pest species but is an extensively studied model organism. The popularity of *D. melanogaster* as a model organism in genetic research has resulted in a fully sequenced and annotated genome and the development of many genetic tools. One of these genetic tools, the *D. melanogaster* Genetic Reference Panel (DGRP), consists of 205 highly inbred lines of *D. melanogaster* each with fully sequenced genomes (Mackay *et al.* 2012). The DGRP allows for the examination of the sex-specific tTA overexpression system in a multitude of differing genetic backgrounds, which are representative of the genetic diversity of a wild population. Utilizing the available DGRP lines and performing a Genome Wide Association Study (GWAS) resulted in the discovery of single nucleotide polymorphisms (SNPs) and linked candidate genes that influence the efficiency of the FL3 system.

Here, we discuss how and why a sex-specific conditional lethal system in *D. melanogaster* varied in efficacy across 201 different genetic backgrounds.

## Materials and Methods

### DGRP crosses/control crosses

FL3#2 was maintained on standard fly diet containing tetracycline at 10 µg/ml. DGRP fly lines were obtained from the Mackay Lab (NC State University) and reared on standard Drosophila diet. Two replicates for each DGRP line were performed, consisting of 10 virgin DGRP females crossed to 10 males from the FL3#2 female lethal line. The flies were transferred to new vials every 3-4 days such that for each cross a total of 4 vials were set. Control crosses were performed on a selection of DGRP lines (14) showing high, low, or moderate female lethality. Two replicates for each selected DGRP line were performed, consisting of 10 virgin DRGP females crossed to 10 Canton-S-B males, the isogenic background of the FL3#2 strain. All crosses were kept in a 25° incubator with 12h light/dark cycles and were fed fly diet without tetracycline. Offspring sex was scored for 20 days after initial setting of the cross. The % female lethality was calculated using the equation [1-(number females/number males)]x100. Mean and standard error were calculated for two replicates per line.

### GWAS

To estimate the broad-sense heritability (*H*^2^) of sex ratio distortion in the offspring of crosses of FL3#2 with the DGRP lines, we used an ANOVA on line means (the average across the two-three replicates for each line). The ANOVA model of form Y=µ+*L*+ε, where Y is average % female lethality, µ is the overall mean, *L* is the random effect of line, and ε is the residual. To identify genetic variants that are associated with sex ratio distortion, the average female lethality per DGRP line served as the phenotypic input for the GWAS using the DGRP2 pipeline developed by the Mackay Lab at North Carolina State University (dgrp2.gnets.ncsu.edu) (Mackay *et al.* 2012; Huang *et al.* 2014). Two significance thresholds were examined (*P* < 10^−6^, *P* < 10^−5^). Candidate variants for functional analysis were selected from both significance thresholds.

### Functional analysis

*Minos* gene disruption lines were obtained from the Bloomington Drosophila Stock Center. Two replicates for each validation experiment were performed, consisting of 10 virgin females from the *Minos* line crossed to 10 FL3#2 males. All crosses were kept in a 25° incubator with 12h light/dark cycles and were fed fly diet without tetracycline. Offspring sex was scored for 20 days to obtain the sex ratio, which was used to calculate the average female lethality per validation line. Average female lethality per line was compared to the corresponding background female lethality.

### Protein-protein network

The list of flybase IDs of genes with GWAS *p* values of less than 10^−5^ were submitted to network analyst (https://www.networkanalyst.ca). A generic protein protein interaction (PPI) was selected with the IrefIndex interactive protein-protein interaction database. A miminum network was selected, which computes the shortest pair-wise paths between all seed nodes.

### RNA isolation and qRT-PCR

Three biological replicates of 10 virgin females crossed to 10 males for each experimental treatment were set on diet containing 0.05% Bromophenol Blue to accurately assess the age of larvae (Maroni and Stamey 1983). Third instar larvae were collected, separated by sex using fluorescence or gonadal development, and 30 per sex per replicate were stored in 1 ml of RNAlater stabilizing solution (Cat#AM7020 Thermo Fisher Scientific Waltham, Massachusetts) at -80° until time of extraction. Total RNA was isolated using the QIAGEN RNeasy mini kit (Cat#74104 Qiagen Venlo, Netherlands) according to manufacturer’s instructions. cDNA was made from the previously isolated total RNA using the Invitrogen SuperScript III First-Strand Synthesis SuperMix kit (Cat#18080-400 Invitrogen) according to manufacturer’s instructions. Random hexamers were used as primers. Reactions containing water instead of enzyme mix were used as negative controls to confirm absence of DNA contamination. To measure relative transcript levels, qRT-PCR was performed with the cDNA template diluted 1:4 with nuclease-free water then pipetted into quadruplicate wells of a 384 well optical plate (Cat#4309849 Applied Biosystems). For each primer set, tTAv and Rpl32 (reference gene), Thermo Maxima SYBR Green/Rox qPCR Master Mix 2X (Cat#K0221 Thermo Fisher Scientific Waltham, Massachusetts) was added to primers to create a master mix, which was then dispensed into wells via a multichannel pipette. The plate was sealed (Cat#4311971 Applied Biosystems), mixed, then centrifuged for 1 min at RT at 1600 g. The qPCR run was performed on a BioRad CFX384 C1000 Touch Thermocycler (BioRad Hercules, California) using the following program: 95° 10 min, [95° 15 s, 60° 60 s, 40x]. Data acquisition was performed on the anneal/extension step. Mean Cq value was found for each set of 4 replicate wells. The reference gene was used to calculate ∆Cq. The tTAv primer pair were tTAv-F (5′ - TCTTGCGTAATAATGCCAAATCCTTCCG - 3′) and tTAv-R (5′ - CCAACACACAGCCCAATGTAAAATGACC - 3′) and the Rpl32 primer pair were Rpl32-F (5′ – ATGCTAAGCTGTCGCACAAATG – 3′) and Rpl32-R (5′ – GTTCGATCCGTAACCGATGT – 3′).

### RNAseq

RNAseq was performed with the same RNA samples used for qRT-PCR with two runs on an Illumina NextSeq 500, which gave 20 million reads per sample. The RNAseq analysis pipeline began with quality checking the reads using FASTQC check (Andrews 2010). Reads were trimmed for adapter and low quality reads were discarded using Trimmomatic (Bolger *et al.* 2014) and the surviving reads were remapped to the *D. melanogaster* genome (version r6.15) (Gramates *et al.* 2017) using Tophat2 (Langmead and Salzberg 2012; Kim *et al.* 2013). Alignments were converted to counts using HTSeq (Anders *et al.* 2015) for differential gene analysis in EdgeR (Robinson *et al.* 2010). The common dispersion for the model was calculated as 0.073424, which was suitable for the negative binomial transformation for the model. Linear contrasts of line means were evaluated to compare the all high lethality DGRP lines *vs.* all low lethality DGRP lines, all high lethality DGRP lines *vs.* low lethality DGRP lines with lower tTA expression, and all high lethality DGRP lines *vs.* low lethality DGRP lines with higher tTA expression for the EdgeR analysis for the DGRP lines. Genes identified as differentially expressed were then grouped using Venn diagram analysis and the intersections were assessed for biological process GO term functional enrichment using the web tool Gorilla (Eden *et al.* 2009), using the entire set of expressed genes as the static reference background.

### Larval fluorescence

Ten virgin DGRP females were crossed to 10 FL3#2 males on standard diet without tetracycline. Female third instar larvae were collected from each cross, rinsed with sterile water, dried, and adhered to white filter paper using double sided tape. Larvae were imaged using a Leica microscope and software. FIJI (ImageJ) imaging software was used to quantify fluorescence for each larva imaged. Fluorescence was normalized using the formula: Corrected Total Fluorescence = Integrated Density – (Area of selected larva * Mean fluorescence of background). Average fluorescence for n larvae (911H n = 8, 153H n = 8, 195H n= 9, 399H n = 16, 492L n= 13, 350L n = 5, 646L n = 8, 317L n = 5) was calculated for each DGRP line.

### Statistical analysis

For the crosses between selected DGRP lines and Canton-S-B (data shown in Figure 2), a one-way random effects model was used. The model may be written Y_ij_ = µ + L_i_ + **ε**_ij_, where L_i_ and **ε**_ij_ denote independent random samples modeling line effects and experimental errors, respectively. If the variance component of line effects is denoted Var(Li)=**σ**_L_^2^, then a test of H0: **σ**_L_^2^ = 0 is not rejected (*i.e.*, the 14 observed line proportions do not differ significantly) and a 95% confidence interval for the overall mean proportion male is (46.4–51.2%). That is, the overall observed proportion male does not differ significantly from 50%. The heritability of sex ratio may be estimated by the intraline correlation coefficient, or ratio of the estimated line variance component to the sum of line and error variance components, 2.25/(2.25+39.88) = 0.0534.

For functional analysis of candidate genes with *Minos* disruption lines, two separate single-factor models were fit with effects for Line. One model was fit for each cross. All pairwise comparisons with the control were carried out using Dunnett’s procedure to control the experiment-wise error rate. The *p* values shown were adjusted for multiplicity.

Associations between measures of tTA RNA and ZsGreen levels were quantified using sample correlation coefficients of residuals from linear models to account for cross effects. Crosses were placed into three groups according to female lethality and tTA RNA levels and pairwise comparisons among these groups were carried out using Tukey’s Honestly Significant Difference.

The most recent version of SAS was used for all statistical analyses unless otherwise noted.

### Data availability

The raw reads were submitted to NCBI-SRA as accessions SAMN08570109-SAMN08570180. All additional information is available as Supplemental material at figshare: https://doi.org/10.25387/g3.11771622.

## Results

### Variation in female lethality across different genetic backgrounds

**Figure 1:**
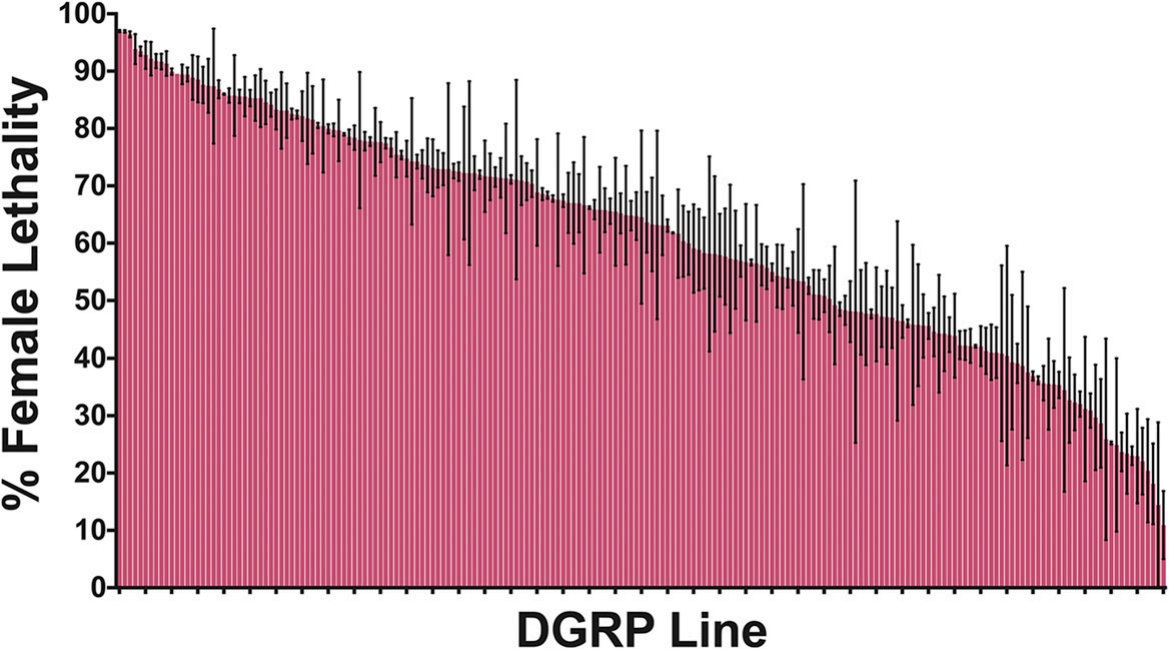
Genetic background strongly influences female lethality. For each cross of a DGRP line with FL3#2, the average percentage of female lethality is represented. The order of DGRP line numbers as presented in this figure can be seen in Supplementary Table 1. Mean and standard error are shown for 2 replicate experiments per DGRP line.

To assess the efficacy of the tTA overexpression conditional lethal system in different genetic backgrounds, we crossed homozygous males from the FL3#2 line to virgin females from 201 of the DGRP lines on standard diet. In the absence of tetracycline, the lethal system is active and most female offspring are expected to die in the late larval/pupal stages due to the overexpression of tTA. The male and female offspring were counted from replicate crosses for each DGRP line to obtain the offspring sex ratio, which was used to calculate the average female lethality per DGRP line. Across the 201 different genetic backgrounds, we saw wide variation (97–11%) in female lethality (Figure 1, Supplementary Table 1). The average female lethality across all DGRP lines was 61%. The DGRP lines showing the highest efficacy of the system (left side of Figure 1) reached 97% female lethality, which is greater than that of FL3#2 heterozygotes in the parental Canton-S-B background (85% female lethality). The DGRP line showing the lowest efficacy (right side of Figure 1) only reached 11% female lethality or 47% female offspring, which approaches the 50/50% sex ratio expected if the lethal system was not present and genetic background did not distort the sex ratio (see below).

**Figure 2:**
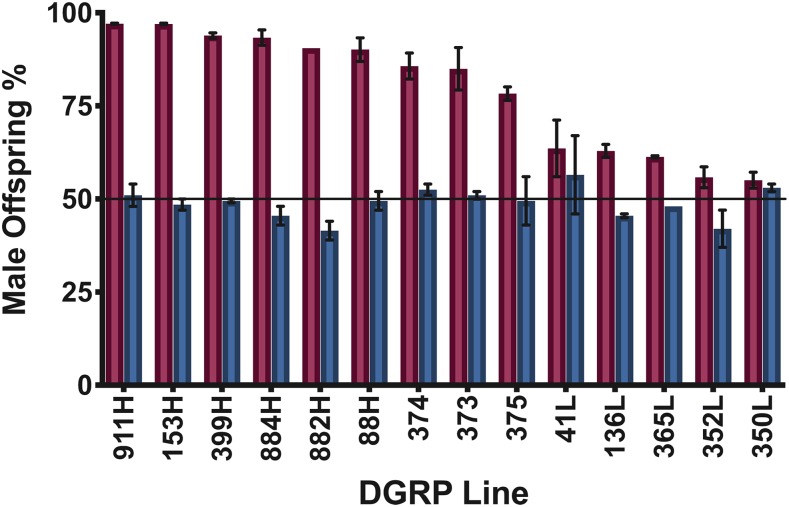
Variation in the observed sex ratios is not due to any inherent sex ratio bias in the DGRP lines. The red bars denote the percentage of offspring that were male in the experimental FL3#2 crosses. The blue bars denote the percentage of offspring that were male in the control crosses of a selected DGRP line with Canton S-B. Mean and standard error are shown for 2 replicate experiments.

It is possible that the observed sex ratio could be skewed by variants present in the DGRP parent. If so, the variant would have a maternal-effect or a dominant effect in the zygote as we are measuring the % female lethality in the offspring of crosses between DGRP females and FL3#2 males. To evaluate if background effects might influence the offspring sex ratio, we chose 6 of the DGRP lines showing the highest female lethality (911, 153, 399, 884, 882, 88), 5 of the DGRP lines showing the lowest female lethality (350, 352, 365, 136, 41), and 3 of the DGRP lines showing intermediate female lethality (374, 373, 375). From these lines, we crossed virgin females to males from Canton-S-B, the isogenic parental line for FL3#2. In all the control crosses, the offspring sex ratio returned to ∼50% for each sex (Figure 2). The observed variability in sex ratio between lines was not significant (*P* = 0.398, F value = 1.15, DF of 14, one-factor random effects model). The heritability of sex ratio was 0.0534. The overall observed proportion male does not differ significantly from 50% (95% confidence interval 46.4–51.2%). Thus, female lethality was due to the tTA overexpression system and not an inherent sex bias among the offspring of any of the DGRP lines (Figure 2).

### Identification of candidate allelic variants

To determine genetic background components that influence the tTA overexpression system, we ran a Genome Wide Association Study (GWAS) using the average percentage of female lethality per DGRP line as the phenotypic input. We saw wide genetic variation in lethality among the DGRP lines with a broad sense heritability of H^2^ = 0.89. The ANOVA results (F = 8.4044, *P* < 0.0001) are shown in Supplementary Table 2. The GWAS output provides both the magnitude of the allelic variant’s effect, through expected correlation with *p*-value, as well as if the variant was associated with lines showing high or low female lethality. The DGRP only has the power to detect associations with large effects (Mackay and Huang 2018). With ∼2.5 million individual tests, the Bonferroni-corrected *p*-value for association is ∼*P* < 2 × 10^−8^ (Mackay and Huang 2018). None of the variants identified in this study were significant at a Bonferroni significance level, which assumes all tests are independent. Further the Quintile-Quintile (Q-Q) plot did not show any variants with observed *p* values lower than random expectation (Fig. S2). However, previous studies using this resource were able to use the DGRP as a screening tool to identify genes for targeted functional analysis using mutations or RNAi knock down of gene expression (Mackay and Huang 2018). Therefore, we proceeded to evaluate the variants with the lowest *p* values. At *P* < 10^−5^ we found 192 candidate allelic variants (FDR =13%) and at a *P* < 10^−6^ we found 16 variants (FDR = 15.6%) associated with either high or low female lethality in the crosses (Supplementary Table 3). Forty-one of these 192 SNPs, insertions, and deletions fell outside of named or annotated genes. However, for those within named genes, 108 different genes were represented with functions ranging from apoptosis to heterochromatin assembly to transcription factor activity (Supplementary Table 3).

### Functional analyses of candidate genes

**Figure 3:**
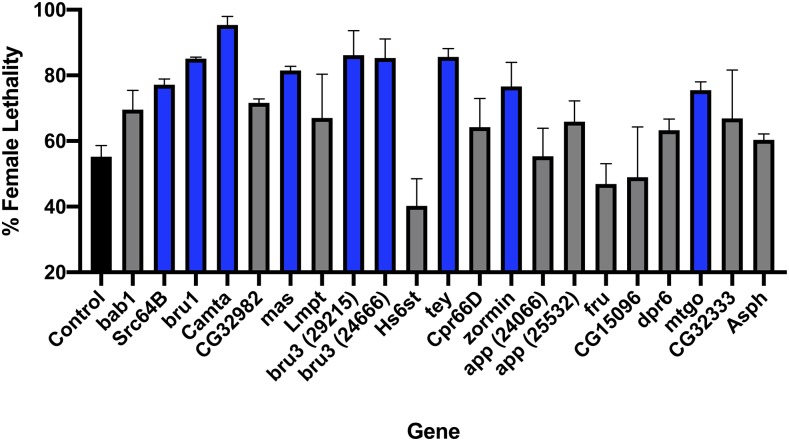
Functional evaluation of candidate genes using gene disruption mutants. All bars show the average percentage of female lethality in offspring of crosses of FL3#2 with the *Minos* disruption mutants for the genes shown. Crosses that showed statistical significance (*P* < 0.05) in the Dunnett’s Test are denoted by blue bars. Cross that were not significant are shown in gray and the control value is represented by a black bar. Mean and standard error are shown for 2 replicate experiments. The genes are shown in the same order as Table 1.

To test whether mutations in genes identified in the GWAS would affect female lethality due to tTA overexpression, we crossed FL3#2 with *Minos* gene disruption lines (Metaxakis 2005) for the genes listed in Table 1. The genes were selected on the basis of *P* values and availability of disruption lines made in an isogenic *w^1118^* strain, to minimize any differences in genetic background between crosses. If the chosen candidate genes influence the tTA overexpression system in some way and the *Minos* insertion disrupts gene function, we expect a change the ratio of female offspring when compared to controls. In the six of the 21 selected lines, the *Minos* element has inserted within an exon (Supplementary Table 4). In the other lines the *Minos* element is within an intron. Virgin females from 21 *Minos* gene disruption lines (2 independent lines were tested for *bru3* and *app*) were crossed to males that carry the FL3 transgene. Female lethality was measured for each of the crosses (Figure 3). To get a baseline for female lethality in the context of the genetic background of each of the knockdown lines, control crosses were performed with the parental isogenic *w*^1118^ strain used to make the *Minos* gene disruption lines (Figure 3). The results from the mutant line crosses were compared to the background control cross results through a Dunnett’s Test for statistical significance (Supplementary Table 4)(Dunnett 1955). Nine of the 21 *Minos* lines tested showed a significant (*P* < 0.05) increase in female lethality compared with the background control. The two independent lines for *bru3* gave similar results with both showing significantly increased female lethality (Figure 3). The two lines for *app* also produced similar results with neither line significantly different than control.

**Table 1:** The candidate genes chosen for functional analysis. *Crosses that were significantly different than their control counterpart using a standard Dunnett’s Test for statistical significance (*P* < 0.05). The genes are listed by p value from the GWAS

Gene	GWAS *p*-value	Function
mas*	5.65E^-06^	serine protease, muscle attachment
CG32982	8.29E^-06^	Unknown function. Predicted role in actin cytoskeleton reorganization
Src64B*	1.01E^-05^	tyrosine-protein kinase,
bru1*	1.02E^-05^	RNA binding, alternative splicing, regulation of translation
Camta*	1.04E^-05^	DNA-binding transcription factor
*dpr6*	1.29E^-05^	Synapse organization, sensory perception
*Hs6st*	1.46E^-05^	Heparin sulfate 6-O-sulfotransferase, imaginal disc growth
*CG32333*	1.55E^-05^	Unknown function, predicted role in lipid metabolism
*bab1*	2.30E^-05^	DNA-binding transcription factor, pigmentation, sex differentiation
tey*	2.32E^-05^	E3 ubiquitin-protein ligase, negative regulation of transcription
mtgo*	2.32E^-05^	Unknown function, essential.
*Asph*	2.78E^-05^	Aspartyl β-hydroxylase that may have a role in neurogenesis
*Cpr66D*	3.0E^-05^	Structural constituent of cuticle
*CG15096*	3.78E^-05^	Transmembrane transport
*app*	4.49E^-05^	Palmitoyltransferase involved in planar cell polarity
zormin*	5.52E^-05^	Protein found in the Z-disc and the M-line of muscles. Likely structural function
*fru*	6.82E^-05^	zinc finger transcription factor important for sexual differentiation
*Lmpt*	7.28E^-05^	Zinc ion binding, defense response
bru3*	7.84E^-05^	RNA binding, negative regulation of translation, regulation of alternative mRNA splicing

#### Protein network:

**Figure 4:**
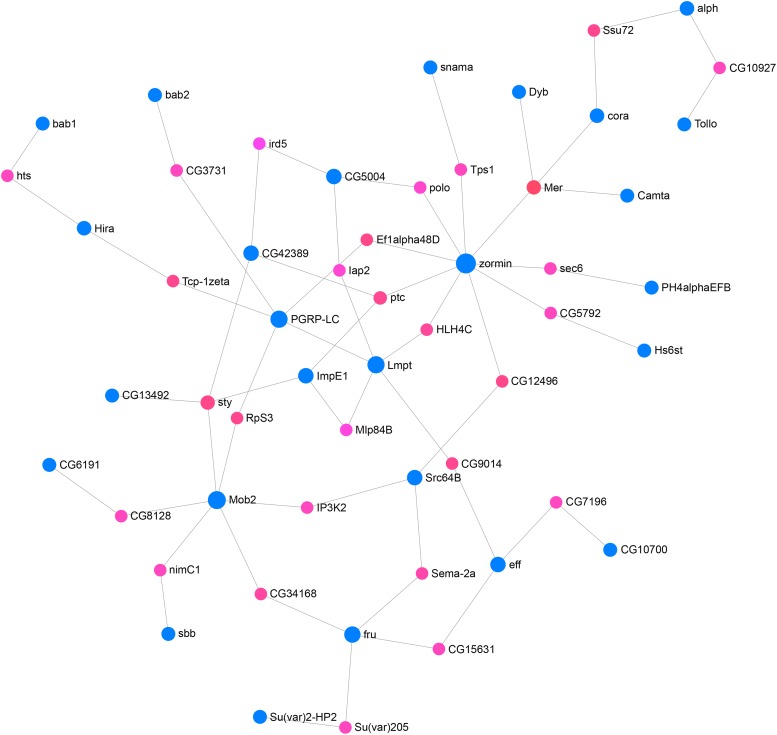
A protein-protein interaction network for variation in female lethality. Candidate genes from GWAS are in blue, other genes are in pink.

We next asked if any of the 108 genes associated with variation in female viability are part of a predicted protein-protein interaction network. Using the IrefIndex interactive protein-protein interaction database we identified a network comprised of 26 interacting candidate genes and 28 computationally recruited genes (Figure 4). Gene ontology analysis showed significant enrichment of several terms associated with cell cycle, nervous system development, regulation of the immune system response and apoptosis (Supplementary Table 5). Six of the 26 candidate genes in the network were functionally evaluated using the *Minos* disruption lines (Figure 3). Three of these genes, *zormin*, *src64B* and *Camta* showed a significant difference in the level of female lethality compared to the parental strain.

### tTA expression levels in different genetic backgrounds

**Figure 5:**
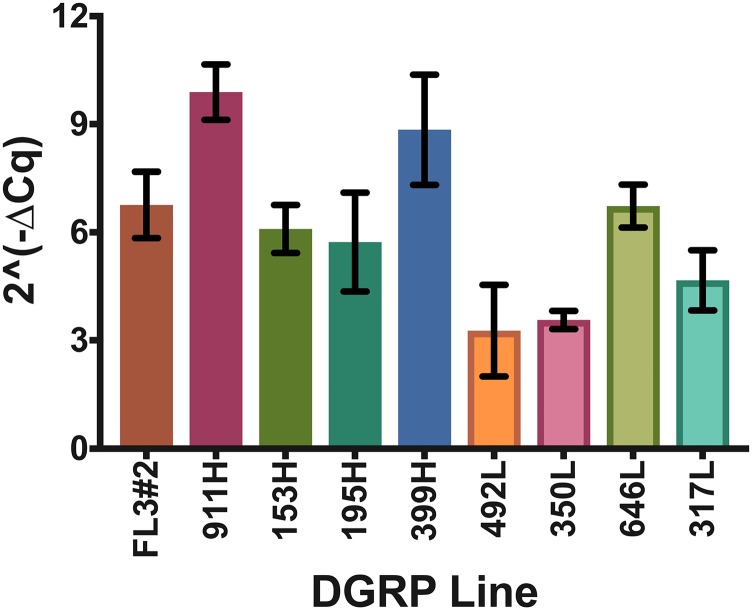
tTA expression in staged female larvae from high and low female lethal backgrounds. tTA expression levels determined by relative transcript abundance for tTA measured by qRT-PCR.

The simplest explanation for high female survival in the low lethality lines is that tTA expression is lower. Observations of the green fluorescence of female larvae collected from the crosses provided the initial indication that tTA was expressed at different levels in some of the lines. This is because larvae that overexpress tTA also show overexpression of the linked fluorescent protein marker gene, ZsGreen (Li *et al.* 2014). This is presumably because tTA bound to *tetO* enhances both the downstream *hsp70* core promoter and upstream *hsp83* promoter that is driving ZsGreen expression (Supplementary Figure 1). Upon closer examination of the larvae collected from the crosses, we found that fluorescence levels were higher in female larvae that showed high lethality compared to low lethal lines (Supplementary Figure 3). To measure tTA RNA levels, we next performed qRT-PCR on RNA from sexed wandering third instar larval offspring from the FL3#2 line crossed with selected DGRP lines. We chose 4 of the DGRP crosses showing high female lethality (911H, 153H, 195H, 399H) and 4 of the DGRP crosses showing low female lethality (492L, 350L, 646L, 317L). For each line, three independent replicates were set on diet without added tetracycline. A blue dye was added to the diet to facilitate accurate staging (Maroni and Stamey 1983). tTA RNA levels were normalized to expression of Rpl32. Female larvae from the crosses that showed high female lethality (911H, 153H, 195H, 399H) all expressed high levels of tTA (Figure 5). The tTA levels were similar or higher than seen in FL3#2. Females from two of the crosses that had low female lethality (492L and 350L) had 1.6 to 3 times lower tTA levels than females from the high lethal crosses (Figure 5). Interestingly, two other crosses that had low female lethality showed higher levels of tTA transcript (646L, 317L), with 646L at the same level seen in FL3#2. Pairwise comparisons among the three groups (high lethal/high tTA [911H, 153H, 195H, 399H], low lethal/high tTA [646L, 317L] and low lethal/low tTA [492L and 350L]) found that tTA RNA levels were significantly different between all groups (Supplementary Table 6). We then returned to larval fluorescence intensity as enhanced ZsGreen expression is an indicator of transcription activation due to tTA bound to the transgene *in vivo*. Third instar larvae of similar size were collected from crosses of FL3#2 with the same DGRP lines used for tTA measurements and were photographed using a green fluorescent filter. FIJI imaging software was used to quantify fluorescence in each larva and average fluorescence per line was determined. There was more variation between replicates than with the qPCR measurements, which reflects the difficulty in quantifying whole-body fluorescence from live larvae. Nevertheless, the highest fluorescence was seen in the larvae from the high lethal/high tTA and low lethal/high tTA groups (Supplementary Figure 3). Further, the high lethal lines had the highest levels of ZsGreen RNA as measured by RNAseq (see below) (Supplementary Figure 3). In pairwise comparisons, the fluorescence intensity and ZsGreen RNA levels of larvae in the low lethal/low tTA group [492L and 350L], were significantly lower than the other two groups (Supplementary Table S6).

### Differential gene expression in genetic backgrounds with low or high female lethality

Differential gene expression was analyzed for the four high female lethal DGRP *vs.* the four low female lethal DGRP used above for the analysis of tTA RNA levels. We used Gorilla (Eden *et al.* 2009) to identify enriched gene ontology (GO) terms in the lists of upregulated and downregulated genes to determine biological processes that are affected by overexpression of tTA. The genes downregulated in the crosses with low female lethality held the strongest enrichment for regulation of Rho mediated signal transduction (FDR < 0.15), embryonic developmental dorsal closure process (FDR < 0.2) and positive regulation of signal transduction (FDR < 0.2) (Supplementary Figure 4). A larger number of biological process GO terms were functionally enriched (*P* < 0.01; FDR < 0.1) among the genes upregulated in the crosses with low female lethality (Supplementary Figure 5). At a FDR of less than 0.05 these included RNA splicing, rRNA processing including modification and protein targeting to the endoplasmic reticulum.

**Figure 6:**
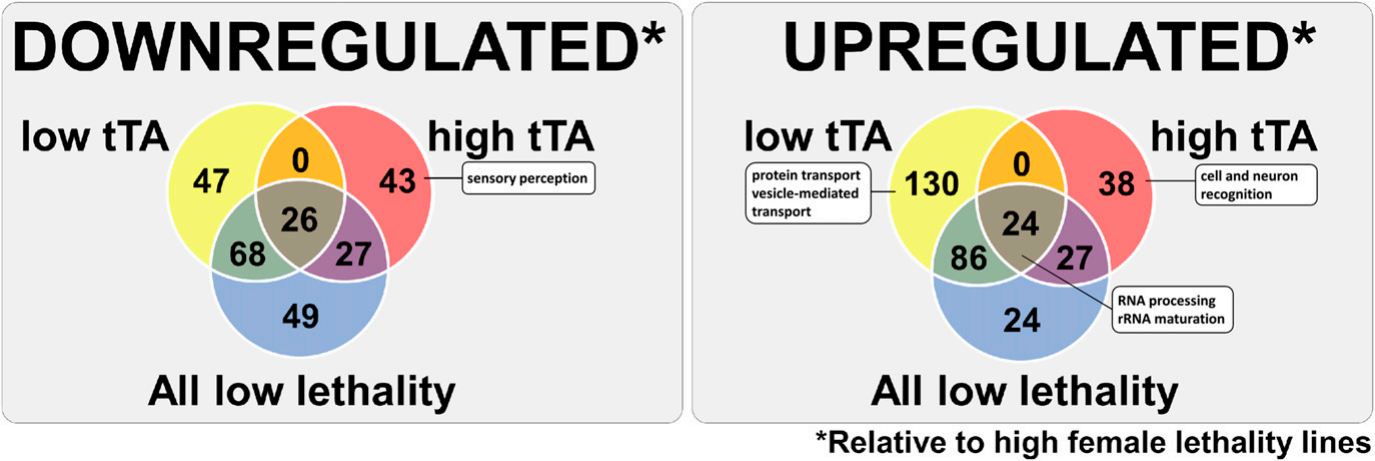
Genes with associated GO terms that are differentially expressed between the high and low female lethality backgrounds. Venn diagram showing the number genes differentially expressed (*P* < 0.01) in all high female lethal DGRP crosses compared with all low female lethal DGRP crosses, all high lethality compared with the low lethality DGRP lines that had either high tTA, or low tTA expression levels.

As lower tTA transcript levels can only partially explain the high female survival observed with some of the crosses, we next compared all of the four high female lethal lines with the two lines that had low lethality and low tTA RNA levels (492L and 350L) using contrast statements in EdgeR (Robinson *et al.* 2010). In addition, all of the high female lethal lines were compared with the two lines that had low lethality but relatively high tTA RNA levels (317L and 646L). One hundred and forty-one genes and 96 genes were downregulated in the low tTA lines and high tTA lines, respectively (*P* < 0.01) (Figure 6 and Supplementary Table 7). Twenty-six of the genes were in common (Figure 6). Sensory perception genes were downregulated in the 317L and 646L crosses. We found no GO term enrichment for the genes in common or downregulated in the low tTA lines. Two hundred and forty genes and 89 genes were upregulated in the low tTA lines and high tTA lines, respectively (*P* < 0.01) (Figure 6 and Supplementary Table 8). Twenty-four genes in common were enriched for the GO terms of RNA processing and rRNA maturation. Genes involved in a number of biological processes, including protein transport and vesicle-mediated transport, were upregulated in the lines with low tTA levels. In the 317L and 646L crosses, genes involved in cell and neuron recognition were upregulated.

**Figure 7:**
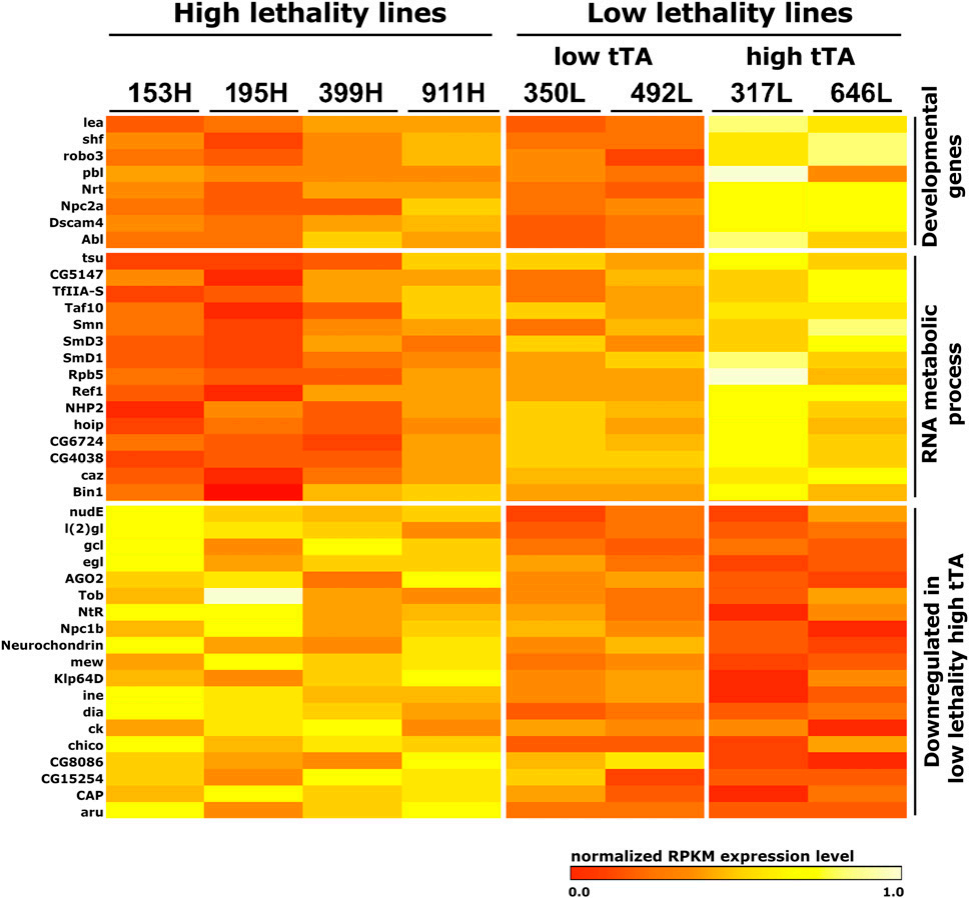
Relative RPKM expression of eight DGRP crosses for selected genes. The genes were associated with functionally enriched biological process GO terms and were identified as differentially expressed between the high female lethality lines and the low female lethality lines that had high tTA expression. RNA was isolated from staged female larvae on diet without tetracycline. RPKM values for a gene (rows) were normalized to the highest RPKM expression level to determine the relative normalized RPKM expression levels across all eight lines.

We next examined the expression levels of the individual genes that were differentially expressed in the lines that had relatively high tTA RNA levels (317L and 646L). We focused on the genes that were associated with the functionally enriched GO terms at the *P* < 0.01, FDR < 0.10 levels of significance. The average RPKM values for each of the 8 crosses were used to plot a heat map (Figure 7 and Supplementary Table 9). Although the RNA splicing and rRNA maturation genes were upregulated in all low lethal lines (Figure 6), it is apparent that expression levels are higher in the low lethal lines with high tTA levels (Figure 7). The genes that were identified as downregulated in the high tTA lines also show somewhat higher levels of expression in the low tTA lines compared to the high lethal lines.

## Discussion

We have shown that the differences in genetic background have a significant effect on the lethality of the tTA overexpression system in *D. melanogaster*. Some backgrounds increased the efficacy of the system in comparison to the developed lethal line (FL3#2) whereas other genetic backgrounds provided almost complete protection from the lethality. These findings are concerning for genetic pest management systems due to the potential that target pest populations may have genetic background components which provide resistance to lethal systems. Similarly, natural variation could influence the efficacy of homing gene drives for population suppression. In crosses of a homing drive with 128 lines from the DGRP collection, it was found that there was significant variation among lines in germline drive conversion and formation of resistance alleles in the embryo (Champer *et al.* 2019).

Through the GWAS, we identified allelic variants in 108 candidate genes and determined their association with increased or decreased efficacy of the tTA overexpression system. However, none of the variants were significant following correction for multiple tests. Further, the Q-Q plot showed the observed *P* value distribution was similar to the expected *P* value distribution under the null hypothesis of no association. This suggests that none of the candidate genes identified influence the tTA overexpression system. However, for most studies using the DGRP resource the *P* values for the majority of variants fall below the Bonferroni-corrected significance threshold (Mackay and Huang 2018). Nevertheless, functional testing of genes associated with variants that did not meet the statistically rigorous threshold (*e.g.*, *P* value <10^−6^) has been a successful strategy for identifying genes that influence a trait (Mackay and Huang 2018). Consequently, we performed crosses of the tTA overexpression line with *Minos* disruption lines and found that 8 of the 19 candidate genes tested had significantly higher female lethality than control. tTA overexpression is thought to cause lethality due to “transcriptional squelching,” that is a general interference in gene expression (Gong *et al.* 2005). Consistent with this hypothesis, some genes identified by the GWAS were involved in gene silencing (*Su(var)2-HP2*) (Shaffer *et al.* 2002; Shaffer *et al.* 2006), chromatin binding (*mamo*) (Hira *et al.* 2013), chromatin remodeling (*Hira*) (Loppin *et al.* 2000) and alternative splicing (*bru1*)(Spletter *et al.* 2015), which could all influence the level of tTA expression. Other candidate genes were involved in defense response (*PGRP-LC*, *Lmpt*)(Jin *et al.* 2008), the septate junction (*cora*) (Tepass *et al.* 2001) and apoptosis (*out*) (Coffman 2003); all of which are systems that could potentially impact survival. Four genes; *eff*, *tey*, *CG32085* and *CG13085*, encode proteins that are predicted to participate in protein ubiquitination and degradation (Thurmond *et al.* 2018; Gramates *et al.* 2017). For example, the Eff protein is a E2 ubiquitin-conjugating enzyme (Chen *et al.* 2009). It has been suggested that overexpression of the tTA protein could cause lethality due to interference with ubiquitin-dependent proteolysis (Gong *et al.* 2005) as ubiquitination of VP16 is required for activity and also signals destruction (Salghetti *et al.* 2001).

We found that 26 of the candidate genes and 28 computationally predicted genes formed a protein-protein interaction network. Crosses with *Minos* disruption strains for three of the candidate genes in the network produced a significantly higher percentage female lethality than control. Gene ontology terms associated with the network included cell cycle, nervous system development, regulation of the immune system response and apoptosis. It would thus be of interest to determine if any of these biological processes are disrupted or enhanced in female larvae that overexpress tTA. However, it should be noted that only a single tTA overexpression strain, FL3#2, has been used in this study. The tTA system is sensitive to position of chromosome integration (Heinrich and Scott 2000; Horn and Wimmer 2003) and thus it is possible that some of the candidate genes may be specific for this strain and not generally involved in the tTA lethal mechanism. For example, a gene product that influences the local chromatin environment of the tTA transgene in the FL3#2 strain may not influence gene expression at other locations in the genome. Further, the set of genes that are over or underexpressed when raised on diet with or without tetracycline appear to be unique for each tTA overexpression strain (Bryk *et al.* 2017).

The simplest explanation for differences in female survival in the different backgrounds is that tTA transcript levels are higher in females from the crosses showing high female lethality. We showed that each of the four selected DGRP crosses that showed high female lethality also expressed high levels of tTA transcript and two of the low female lethality lines showed tTA levels that were significantly lower. This would be consistent with the GWAS analysis that identified variants linked with genes that influence transcription. The high female survival in two lines that had relatively high levels of tTA (317L and 646L), suggests that there may be other mechanisms that provide resistance to the otherwise lethal consequences of tTA overexpression. To gain an insight into how differences in gene expression could influence female survival, we performed RNAseq on sexed late stage 3^rd^ instar larvae. We searched for enrichment of GO terms in the differentially expressed genes. We found genes important for protein transport, vesicle transport, hemostasis and RNA processes were overexpressed in the low lethal lines. Similarly, genes important for embryo development and signal transduction were downregulated. Genes involved in RNA metabolic processes included transcription, mRNA splicing and rRNA maturation were overexpressed in all of the low lethal lines but particularly in the 317L and 646L lines. It is possible that higher expression of these genes could counteract the general interference with gene expression due to high levels of tTA. Three of the genes encode proteins important for transcription initiation (*TfIIS*, *Taf10* and *caz*) and a fourth protein, *Rpb5*, is a RNA polymerase II subunit (Aoyagi and Wassarman 2000). The proteins encoded by the mRNA processing genes associated with low female lethality include spliceosomal snRNP proteins (*SmD1*, *SmD3*), *smn*, which interacts with snRNPs (Kroiss *et al.* 2008), *tsu*, a component of the exon-junction complex and components of other spliceosomal complexes (*caz*, *hoi*, *Bin1*) (Herold *et al.* 2009). The rRNA processing genes are involved in pseudouridylation (*CG4038* and *NHP2*). RNA modifications generally stabilize the ribosome but may also contribute to translational control of gene expression (Sloan *et al.* 2017). Genes important for essential developmental processes were also overexpressed in the 317L and 646L lines. These include genes important for axon guidance (*robo2*, *robo3*, *Dscam4*, *Nrt*) and signal transduction (*abl kinase*, *pbl* and *shf*). The downregulated genes in the 317L and 646L lines were enriched for GO terms associated with sensory perception and nervous system processes. It is not readily apparent how decreased expression of these genes would increase female survival. These experiments show that resistance to the tTA overexpression lethal mechanism could arise in a target pest population due to preexisting genetic variation that either provides protection by directly impacting the level of tTA transcript produced or by mitigating the otherwise lethal consequences of high tTA levels. Further, there is a need for more fundamental studies on the mechanism of lethality due to tTA overexpression and the potential role(s) of the candidate genes identified in this study.

Resistance in a target pest population could potentially be avoided by releasing strains that combine dual redundant lethality systems that do not share components (Eckermann *et al.* 2014; Handler 2016). For example, engineer a strain that has a tetracycline-repressible female lethal system and an independent temperature-sensitive lethal system (Handler 2016). Combining multiple systems decreases the likelihood that a pest population would become resistant to all components. Another method for reducing the risk of resistance is to use a single transgene that shows 100% dominant lethality when tested in multiple different genetic backgrounds (Concha *et al.* 2016). The fully penetrant *C. capitata* fsRIDL strains combined two tTA overexpression systems into a single transgene construct (Leftwich *et al.* 2014), which could also reduce the risk of resistance arising in the target population. The spread of resistance in the target population could be limited by introgression of susceptible alleles from the continued release of homozygous males carrying a dominant female lethal system (Alphey *et al.* 2011). However, subsequent analysis using a spatial model found that interactions between the target population and insects in a non-target area can produce a range of outcomes from enhancing genetic suppression to accelerating the proliferation of resistance (Watkinson-Powell and Alphey 2017).

We have shown that variation in genetic background components can lead to both strengthening and weakening a lethal system. While *D. melanogaster* is not itself an economic pest species, many of the genes identified in this study have orthologs in species that are pests. We advise evaluation of any insect strain that relies solely on tTA overexpression as the lethal mechanism by crossing with strains collected from the location of the target pest population.
